# Synthesis, Characterization, and Stability Study of Selenium Nanoparticles Coated with Purified Polysaccharides from *Ononis natrix*

**DOI:** 10.3390/nano15060435

**Published:** 2025-03-12

**Authors:** Nour Bhiri, Nathalie Masquelez, Moncef Nasri, Rim Nasri, Mohamed Hajji, Suming Li

**Affiliations:** 1 Institut Européen des Membranes, IEM, UMR 5635, University of Montpellier, CNRS, ENSCM, 34095 Montpellier, France; nour.bhiri@enis.tn (N.B.); nathalie.masquelez@umontpellier.fr (N.M.); 2 Laboratory of Enzyme Engineering and Microbiology, National School of Engineering of Sfax (ENIS), University of Sfax, P.O. Box 1173, Sfax 3038, Tunisia; mon_nasri@yahoo.fr (M.N.); rymnasri2@gmail.com (R.N.)

**Keywords:** selenium nanoparticles, *Ononis natrix*, polysaccharide, stabilization, antioxidant activity

## Abstract

Selenium nanoparticles (SeNPs) attract considerable attention for their promising applications in the biomedical field, driven by their unique properties and antioxidant activities. However, their practical use is often hindered by issues such as instability and aggregation. In this study, a polysaccharide, P2, extracted from *Ononis natrix*, was used to stabilize SeNPs to address these limitations. P2-SeNPs were prepared through a green synthesis method involving sodium selenite, P2, and ascorbic acid, and characterized by dynamic light scattering (DLS), transmission electron microscopy (TEM), Fourier-transform infrared (FT-IR) spectroscopy, and X-ray diffraction (XRD). P2-SeNPs exhibited a smaller particle size and enhanced stability compared to unmodified SeNPs. UV-Vis spectroscopy and X-ray photoelectron spectroscopy (XPS) demonstrated the presence of Se–O bonds, suggesting effective stabilization by covalent bonding between SeNPs and P2. Stability tests revealed that P2-SeNPs maintained good dispersion under various conditions, with optimal stability observed at refrigerated temperatures and neutral pH. Moreover, P2-SeNPs exhibited better antioxidant activities than unmodified SeNPs, as evidenced by higher DPPH radical scavenging, ABTS radical scavenging, and metal chelation ratios. This difference is attributed to both the reduced aggregation and smaller size of P2-SeNPs. Therefore, it is concluded that P2-SeNPs exhibit significant potential as an effective antioxidant agent for biomedical applications.

## 1. Introduction

In recent years, advancements in nanotechnology have profoundly influenced drug development and the field of nanomedicine [1]. Metal nanoparticles have emerged as pivotal elements in nanotechnology, offering great potential as carriers for chemotherapeutic drugs, proteins, and siRNA, etc. Among them, selenium nanoparticles (SeNPs) have garnered particular attention due to their unique biological properties. Selenium (Se) was first identified by Jöns Jacob Berzelius in 1817 [2]. The element was named after the Greek word ‘Selene,’ meaning moon [3]. In its elemental form, selenium is colorless, non-toxic, and biologically inert [2]. It plays a crucial role in maintaining a healthy immune system and preventing disease [4]. It is also involved in cancer prevention, cardiovascular health, and alleviating fatigue [5,6,7]. Recent research has emphasized the benefits of SeNPs as an innovative selenium-based material, particularly in terms of their high biocompatibility and surface modifiability compared to other selenium compounds, which has sparked significant interest [8,9]. However, a major challenge with SeNPs is their instability and tendency to agglomerate, resulting in black precipitates that greatly diminish their biological accessibility [10]. Thus, improving the stability of SeNPs has become a research focus. To address this issue, various biological macromolecules, including polysaccharides [11], peptides [12], and synthetic polymers [13], have been utilized to functionalize SeNPs, preventing aggregation and improving their stability for enhanced biological effectiveness.

Polysaccharides derived from natural sources, including plants, animals, fungi, and marine algae [14], have been widely investigated in biotechnology and biomedical applications [15], due to their distinctive characteristics, such as biocompatibility and biodegradability, which are essential for biomaterial applications [16]. Polysaccharides, when utilized as stabilizing and dispersing agents, have notably enhanced the stability, biocompatibility, and biological properties of SeNPs. The antioxidant characteristics of polysaccharide-coated SeNPs have gained significant interest, as oxidative damage is a key factor in various diseases and injuries [17].

*Ononis natrix* L. (Leguminosae/Fabiaceae) is a perennial shrub native to Africa, Southwestern Europe, and the northwest region of Saudi Arabia. This plant has been traditionally used to treat urinary tract infections due to its diuretic, blood pressure-lowering, antibacterial, and anti-inflammatory properties [18]. Importantly, polysaccharides extracted from *O. natrix* exhibit notable antioxidant and antibacterial activities [19]. However, there is currently no information available regarding the use of *O. natrix* polysaccharides as stabilizers for SeNPs.

In this study, polysaccharides obtained from *O. natrix* were purified and characterized. The purified polysaccharides, namely P2, were then utilized as stabilizing and capping agents to synthesize SeNPs using a redox system composed of sodium selenite (Na_2_SeO_3_) and ascorbic acid known as Vitamin C. The resulting P2-SeNPs were characterized by using various techniques, including dynamic light scattering (DLS), transmission electron microscopy (TEM), Fourier-transform infrared (FT-IR) spectroscopy, UV-Vis absorption spectroscopy (UV-Vis), X-ray diffraction (XRD), and X-ray photoelectron spectroscopy (XPS). The stability of P2-SeNPs was assessed under different conditions. Furthermore, the in vitro antioxidant activity of P2-SeNPs was examined and compared to that of SeNPs.

## 2. Materials and Methods

### 2.1. Materials

Polysaccharides were extracted from *O. natrix* using hot water maceration method. More detailed characterization of obtained polysaccharides has been reported in our previous paper [19]. The 1,1-diphenyl-2-picrylhydrazyl (DPPH) and 2,2′-azino-bis (3-ethylbenzothiazoline-6-sulfonic acid) (ABTS), sodium selenite, and ascorbic acid were purchased from Sigma Chemical Co. (St. Louis, MO, USA). All other chemicals were of analytical grade and used as received.

### 2.2. Purification of Polysaccharides Extracted from O. natrix

The crude polysaccharide was dissolved in deionized water and centrifuged at 5000 rpm for 20 min. The supernatant, containing soluble polysaccharides, was applied to a DEAE-Sepharose Fast Flow column (25 mm × 30 cm) and eluted with 100 mL of water, followed by elution with of NaCl solutions with varying concentrations of 0.2, 0.4, and 0.6 M at a flow rate of 3 mL/min, and a volume of 5 mL for each collected aliquot. The eluate fractions were collected and analyzed using the phenol-sulfuric acid method. The primary fraction eluted with water was collected and lyophilized, resulting in purified polysaccharides designated as PS1. Likewise, fractions eluted with 0.2 and 0.4 M NaCl were collected and lyophilized. PS1 was further purified by gel filtration chromatography using a Sephadex G-100 column (Ø 1.5 × 100 cm). Distilled water served as the mobile phase, applied at a flow rate of 0.5 mL/min, and monitored with the phenol-sulfuric acid method. Fractions corresponding to P1 and the main peak, P2, were collected and lyophilized. The carbohydrate content was determined using the phenol-sulfuric acid method, with D-glucose as the standard [20].

### 2.3. Synthesis of SeNPs Stabilized with Purified Polysaccharides P2 (P2-SeNPs)

P2-SeNPs were prepared by reducing sodium selenite with ascorbic acid. In brief, P2 solutions at concentrations ranging from 1 to 5 mg/mL were mixed with an equal volume of 0.01 M Na_2_SeO_3_ solution under magnetic stirring. Afterward, an equal volume of freshly prepared 0.04 M ascorbic acid solution was added dropwise to the mixture under vigorous stirring and the reaction mixture was maintained at 40 °C in the dark for 4 h. The pH of the reaction medium was around 4.2. Ultimately, the solution was dialyzed (MWCO 3500 Da) against deionized water in the dark at 4 °C for 3 to 4 days to remove excess ascorbic acid and Na_2_SeO_3_. For comparison, SeNPs were prepared using the same procedure, but with an equal volume of distilled water substituted for the P2 solution.

### 2.4. Physicochemical Characterization

#### 2.4.1. Gel Permeation Chromatography

The molecular weight of polysaccharides was assessed using the gel permeation chromatography (GPC). Measurements were made at 30 °C on a ‘Shimadzu Nexera LC system’ equipped with two detectors: RID-20A Refractive Index Detector and SPD-40 UV detector. The system is equipped with three columns: 1 × PL Aquagel-OH 8 µm guard column (50 × 7.5 mm, Agilent, Santa Clara, CA, USA), 1 × PL Aquagel-OH 30 8 µm (300 × 7.5 mm, Agilent), and 1 × PL Aquagel-OH 40 8 µm (300 × 7.5 mm, Agilent). The mobile phase was a pH 7 phosphate buffer and the flow rate was 1 mL/min. Pullulan standards were used for calibration.

#### 2.4.2. Nuclear Magnetic Resonance

Proton nuclear magnetic resonance (^1^H NMR) and carbon nuclear magnetic resonance (^13^C NMR) spectra were recorded at 25 °C on a Bruker Avance III spectrometer (Bruker, Billerica, MA, USA). Deuterium oxide (D2O) served as the solvent. Chemical shifts are reported in parts per million (ppm). The spectra were analyzed using Mestre Nova 5.3.0 software from Mestrelab Research (Santiago de Compostela, Spain).

#### 2.4.3. Fourier-Transform Infrared (FT-IR) and UV-Visible Spectroscopy

FT-IR analysis was conducted using a Cary 630 series spectrometer (Agilent Technologies) fitted with an attenuated total reflection (ATR) accessory containing a diamond/ZnSe crystal. An average of 32 scans were recorded with a resolution of 4 cm^−1^ over the wavenumber range of 650–4000 cm^−1^. Data analysis was carried out using OMNIC 9.16 Spectra software (Thermo Fisher Scientific, Waltham, MA, USA). A UV-visible spectrophotometer was employed to measure ultraviolet absorbance of solutions in the range of 200 to 800 nm. Measurements were made directly from the solutions of synthesized SeNPs and P2-SeNPs, and from a P2 solution at a concentration of 1.3 mg/mL.

#### 2.4.4. Thermogravimetric Analysis (TGA)

TGA thermograms were recorded using a TGAQ 500 Analyzer (TA Instruments, New Castle, DE, USA). Measurements were carried out within a temperature interval of 25 °C to 700 °C with a heating rate of 20 °C/min.

#### 2.4.5. Dynamic Light Scattering (DLS)

The average particle size and zeta potential were measured using dynamic light scattering (DLS) using a Litesizer 500 particle instrument (Anton Paar, GmbH, Les Ulis, France) at 25 °C. Measurements were performed in triplicate using the solutions of synthesized nanoparticles.

#### 2.4.6. Transmission Electron Microscopy (TEM)

TEM analysis was conducted using a 200 kV TEM 2200FS instrument (JEOL, Akishima, Japan). P2-SeNPs and SeNPs samples were prepared by depositing a drop of 5 µL onto a Formvar/carbon-coated Cu grid, followed by air drying at ambient temperature before measurements. TEM images were analyzed using ImageJ software 1.54 (National Institutes of Health, Bethesda, MD, USA).

#### 2.4.7. X-Ray Diffraction (XRD)

XRD patterns were recorded with an X-ray diffractometer (Bruker D5000) with a Cu-Kα radiation source. Measurements were carried out from 7 to 40° (2θ, diffraction angle) at a scanning speed of 1°/min. The diffractograms were obtained at a voltage of 40 kV and a current of 20 mA.

#### 2.4.8. X-Ray Photoelectron Spectroscopy (XPS)

XPS spectra were recorded on a Thermo Electron ESCALAB 250 spectrometer to determine the valence state of selenium in lyophilized NPs, using the Al Kα line (1486.6 eV) as a monochromatic excitation source. The photoelectrons were assessed with the sample surface at normal incidence. A surface area of 500 µm^2^ was examined. The photoelectron spectra were calibrated for binding energy relative to the C-C component of the carbon 1 s peak at 284.8 eV. Charge compensation was achieved by employing an electron beam (−2 eV).

### 2.5. Stability Test

The storage stability was investigated by monitoring the effects of light, pH, ionic strength, temperature (4 and 25 °C), and duration (0 to 30 days) on P2-SeNPs. DLS measurements were performed at regular intervals to determine the average particle size of P2-SeNPs in solution.

To evaluate the impact of pH on stability, the pH of P2-SeNPs solutions was adjusted to 2, 7, and 10 using 0.1 M HCl/NaOH and maintained for 1 h before measuring particle size.

The stability under different ionic strengths was evaluated by adding NaCl solution (2 mL) at various concentrations (10, 50, 100, 200 mM) to the P2-SeNPs solution (2 mL). The particle size was determined after 1 h stirring.

### 2.6. Antioxidant Activities

The DPPH radical scavenging activity was evaluated following the method described by Bersuder [21]. A freshly prepared nanoparticles solution (500 µL) was combined with 500 µL of DPPH solution (0.2 mM in ethanol). After incubation in complete darkness at 25 °C for 30 min, the absorbance of the mixture was measured at 517 nm. The radical scavenging activity was calculated using the following equation:

Radical scavenging activity (%) = (A_c_ + A_b_ − A_e_)/A_c_ × 100 (1)

where A_c_, A_b_, and A_s_ represent the absorbance of the control (containing all reagents except the sample), the blank (containing all reagents except the DPPH solution), and the sample solution in the reaction mixture, respectively.

The ferrous chelating capacity was determined using the method of Carter [22]. Firstly, 100 mL of 2 mM FeCl_2_ were mixed with 200 mL of freshly prepared nanoparticles solution. After incubating for 5 min at room temperature, 400 µL of a 5 mM ferrozine solution was introduced into the mixture. The solution was then vigorously stirred and the reaction was allowed to proceed for 10 min at room temperature. Finally, the absorbance was recorded at 562 nm. The ferrous ion-chelating activity, representing the inhibition of ferrozine/Fe^2^⁺ complex formation, was calculated using the following equation:

Ferrous ion-chelating activity (%) = (A_c_ + A_b_ − A_e_)/A_c_ × 100 (2)

where A_c_ corresponds to the absorbance of the control (without the sample), A_b_ represents the absorbance of the blank (without ferrozine), and A_e_ is the absorbance of the sample. Each measurement was performed in triplicate to ensure accuracy.

The total antioxidant activity of a molecule is assessed based on its ability to inhibit the ABTS radical cation (ABTS^•+^), generated from ABTS. To prepare the ABTS^•+^ solution, a 7 mM aqueous solution of ABTS was mixed with a 2.45 mM aqueous solution of potassium persulfate in equal volumes and kept in the dark at room temperature for 12 h. Subsequently, an aliquot (3 mL) of the ABTS^•+^ solution was added to a freshly prepared nanoparticle solution (1 mL) and incubated in the dark at 25 °C for 30 min. The absorbance at 734 nm was then recorded using a UV-visible spectrophotometer. The ABTS radical scavenging activity was determined using the following equation:

ABTS radical scavenging activity (%) = (A_c_ + A_b_ − A_e_)/A_c_ × 100 (3)

where A_c_ corresponds to the absorbance of the control (containing all reagents except the sample), A_b_ represents the absorbance of the blank (containing all reagents except the ABTS radical cation solution), and A_e_ is the absorbance of the sample in the reaction mixture [23].

### 2.7. Statistical Analysis

Statistical analysis was conducted using SPSS version 17.0 (professional edition). Mean differences were assessed using the Duncan test and compared via one-way analysis of variance (ANOVA). A *p*-value < 0.05 was considered statistically significant. All experiments were performed in triplicate.

## 3. Results

### 3.1. Purification of O. natrix Polysaccharides

**Figure 1:**
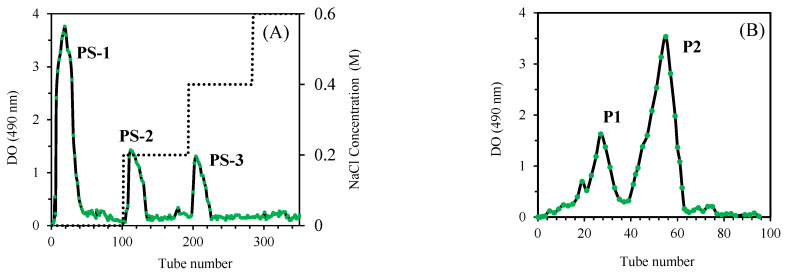
Elution profile of PS on a DEAE-Sepharose Fast Flow column (25 mm × 30 cm), eluted with 100 mL of deionized water and a NaCl solution applied as a step gradient from 0.2 to 0.6 M (dotted line), at a flow rate of 3 mL/min, with 5 mL aliquots collected for each fraction (**A**). Elution profile of freeze-dried PS-1 polysaccharides on a Sephadex G-100 gel filtration column, eluted with deionized water (**B**).

The purification of crude polysaccharides was carried out using two complementary chromatography techniques: ion-exchange chromatography and gel filtration chromatography. The first purification step involved ion-exchange chromatography on a DEAE-Sepharose fast flow column. This technique allowed for the separation of three fractions: PS-1, which was eluted with deionized water, and PS-2 and PS-3, which were eluted at NaCl concentrations of 0.2 M and 0.4 M, respectively. The yield obtained for these fractions was 61.2 ± 1.7% for PS-1, 11.9 ± 0.5% for PS-2, and 6.8 ± 0.7% for PS-3 (Figure 1A). The PS-1 fraction, which corresponded to the main peak observed during the initial separation, underwent additional purification via gel filtration chromatography using a Sephadex G-100 column. Gel filtration chromatography allowed the isolation of two purified fractions, named P1 and P2, by eluting with deionized water. The yield of P1 and P2 was 17.9 ± 0.6% and 67.5 ± 1.3%, respectively. Analyses revealed a total sugar content of 94.5 ± 0.3% and 98.1 ± 0.5% for P1 and P2, respectively, indicating a very high purity of the obtained polysaccharides (Figure 1B).

### 3.2. Structural Characterization of Purified Polysaccharides

#### 3.2.1. Molecular Weights

GPC was employed to assess the molecular weights of the purified fractions P1 and P2, using pullulan as the calibration standard. As illustrated in Table 1 and Figure S1 (Supplementary Data), P1 exhibits two distinct fractions, a small fraction with weight-average molecular weight (Mw) of 732.6 kDa and dispersity (Ð) of 1.1, and a major fraction with Mw of 74.4 kDa and Ð of 1.6. The presence of two peaks suggests that P1 consists of chains of varying lengths. In contrast, P2 displays a single peak with Mw of 30.2 kDa and Ð of 1.7. Although P2 shows slightly higher dispersity and lower average Mw, the absence of multiple fractions suggests higher homogeneity in chain lengths compared to P1.

#### 3.2.2. FT-IR Analysis

**Figure 2:**
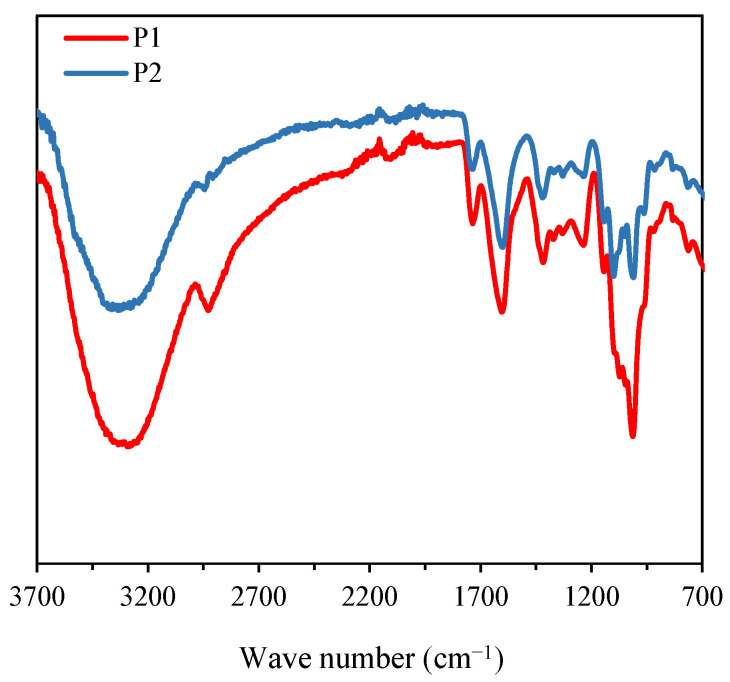
FT-IR spectra of purified polysaccharides P1 and P2.

Figure 2 displays the FT-IR spectra of the purified polysaccharides, namely P1 and P2. Both polysaccharides exhibit characteristic bands of hydroxyl groups (O-H) at approximately 3325 cm^−1^ and C-H stretching vibrations around 2929 cm^−1^ [24]. P1 and P2 also present C=O stretching bands at 1741 cm^−1^, as well as C-O stretching bands at 1417 cm^−1^, and asymmetric C=O or C-O stretching vibrations at 1606 cm^−1^ [25]. The signal at 1267 cm^−1^ is associated with C-O stretching vibrations [26]. The absorption peaks at 1097 cm^−1^ and 1010 cm^−1^ are attributed to the vibrations of the C-O-C group in a six-membered ring [25]. In summary, P1 and P2 present similar functional groups, but the band at 1097 cm^−1^ reveals some structural difference between the two polysaccharides.

#### 3.2.3. NMR Analysis of Purified Polysaccharides

**Figure 3:**
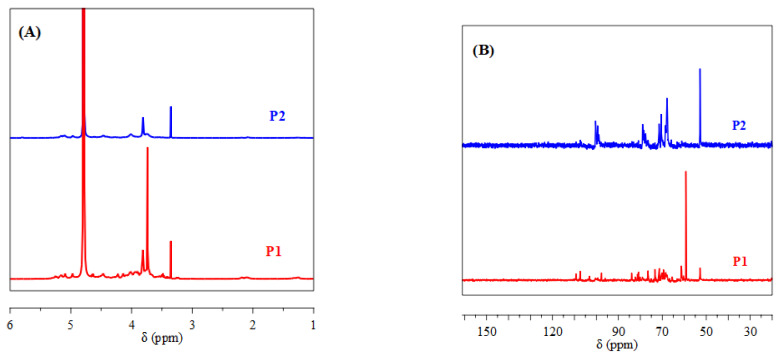
^1^H NMR (**A**) and ^13^C NMR (**B**) spectra of P1 and P2.

P1 and P2 were analyzed by NMR to gain further insight into their structures and chemical compositions. As shown in Figure 3A, the hydrogen signals (H-2 to H-6) of P1 and P2 are concentrated in the δ 4.5–3.0 ppm region, characteristic of polysaccharides. The signals between 4.2 and 4.7 ppm and 4.8 and 5.2 ppm correspond to the α-anomeric and β-anomeric protons, respectively, indicating the presence of both α- and β-terminal configurations in both polysaccharides [27]. A prominent signal at 5.10 ppm, attributed to H-1 of α-Rha residues, is detected in the spectra of both P1 and P2 [28]. Signals observed at 4.47 ppm in P1 and 4.46 ppm in P2 indicate β-(1→3)-glycosidic linkages [29]. The region between 3.2 and 3.77 ppm is characteristic of polysaccharide residues, including galactose and xylose [30]. Signals at 3.82 ppm in both samples belong to β-D-Galp residues [31].

The ^13^C NMR spectra reveal the presence of α and β configurations for the anomeric carbons, with chemical shifts in the ranges of 90–100 ppm and 100–106 ppm (Figure 3B), respectively [32]. The 69–79 ppm region is identified as the pyranose ring region (C2–C5) [33]. Signals at 107 ppm in P1 and 84 ppm in P2 are related to the presence of furanose rings [34]. Signals in the 5.50–4.40 ppm (^1^H NMR) and 90–110 ppm (^13^C NMR) regions are assigned to the anomeric protons and carbons of D-Galp, L-Rhap, and D-Araf residues, respectively [35,36]. Signals at 78.6 and 71.3 ppm in P1 and P2 are attributed to L-Rhap carbons, while signals around 68 ppm correspond to C-6 of D-Galp [37]. Signals recorded at 103.1 and 61.4 ppm in P1 for galactoses and at 100.4 ppm in P2 for xylose are also identified [38]. Finally, signals at 107.3 ppm in P1 and P2 correspond to α-L-Araf [39]. P1 and P2 polysaccharides reveal common structural features, such as the presence of α-L-Rhamnose residues, β-(1→3)-glycosidic linkages, and β-D-Galactose residues. However, differences may exist in the relative proportions of these units.

#### 3.2.4. Thermogravimetric Analysis of Purified Polysaccharides

The TGA thermograms of polysaccharides P1 and P2 are shown in Figure S2. Up to 200 °C, a mass loss of 17% and 20% is detected for P1 and P2, respectively, which is attributed to the evaporation of adsorbed water, including both free water and bound water [40]. Beyond this temperature, between 200 °C and 400 °C, a major decomposition of polysaccharides occurs. P1 decomposes more extensively than P2, indicating a significant weight loss. The third stage occurs beyond 450 °C, where both P1 and P2 undergo continuous decomposition with increasing temperature [41], leading to further weight loss. A total weight loss of 83.6% and 71.3% is obtained for P1 and P2, respectively. It is suggested that the significant degradation of P1 during the third stage may be due to its predominantly linear polysaccharide structure. Earlier research has indicated that a highly branched polysaccharide structure can notably improve thermal stability [42].

### 3.3. Synthesis and Characterization of P2 Stabilized Selenium Nanoparticles

By combining selenium nanoparticles (SeNPs) with natural polysaccharides using a green strategy, the inherent limitations of SeNPs can be effectively overcome, thereby enhancing their bioavailability and expanding their potential applications in biomedicine. In this work, purified polysaccharide P2 was chosen as the stabilizer for SeNP synthesis due to its high yield and molecular weight uniformity. P2-functionalized SeNPs (P2-SeNPs) were synthesized by reacting sodium selenite with ascorbic acid.

#### 3.3.1. Particle Size and Dispersion

**Figure 4:**
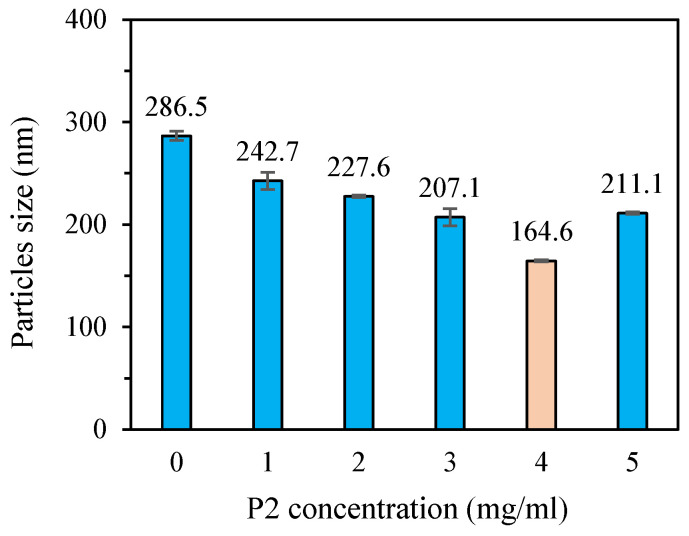
Particle size variation of SeNPs prepared using P2 as stabilizer at various concentrations.

DLS is an effective technique for assessing the particle size, size distribution, and zeta potential of nanomaterials in solution. To obtain nano-sized SeNPs with good stability, five types of SeNPs stabilized with P2 concentrations from 1 to 5 mg/mL, along with a control without P2, were prepared. The presence of P2 significantly influenced (*p* < 0.05) the average particle diameter (Figure 4). In the SeNPs solution without P2, the selenium is unstable and tends to aggregate. A diameter of 286.5 nm was observed. Upon the addition of P2 to the reaction mixture, polysaccharide chains were able to adsorb onto the surface of the growing SeNPs due to the abundance of hydroxyl groups, which resulted in improved dispersion and a significant reduction in particle size [43,44]. The diameter decreases with increasing P2 concentration in the reaction mixture, reaching 242.7, 227.6, 207.1, and 164.6 nm for P2-SeNPs at a P2 concentration of 1, 2, 3, and 4 mg/mL, respectively. However, at a concentration of 5 mg/mL, the particle size of P2-SeNPs increased to 211.1 nm. This can be attributed to the interactions between excess P2 chains present in the solution. Indeed, the interactions between P2 chains are stronger than those between P2 and SeNPs, leading to agglomeration of the SeNPs because of less P2 chains available for stabilization [45]. In conclusion, at a P2 concentration of 4 mg/mL, P2-SeNPs exhibit the smallest particle size. Further analyses will be conducted on P2-SeNPs prepared with P2 at a concentration of 4 mg/mL.

As illustrated in Figure S3A, the SeNPs solution prepared without using P2 as stabilizing agent is turbid due to aggregation. In contrast, the addition of P2 (4 mg/mL) results in a clear orange-red solution of P2-SeNPs (Figure S3A). It is assumed that the presence of polysaccharides protects SeNPs from oxidation during lyophilization. The protective layer provided by P2 likely inhibits interaction of selenium with oxygen, maintaining the integrity of the nanoparticles (Figure S3B). Additionally, SeNPs without P2 tended to precipitate, whereas the orange-red solution of P2-SeNPs remained stable and homogeneous, with no visible precipitation after 30 days of storage at 4 °C (Figure S3C). These findings indicate that P2 plays a crucial function in stabilizing SeNPs. According to Stokes’ law, systems with larger particle sizes exhibit lower stability [46]. Moreover, P2-SeNPs exhibit a more negative zeta potential (−13.8 mV) compared to SeNPs (−6.3 mV), demonstrating improved stability of P2-SeNPs in aqueous solution. All these findings suggest that P2 at an optimal concentration (4 mg/mL) plays a crucial role in stabilizing SeNPs. Moreover, it is worth mentioning that SeNPs present a more uniform particle size distribution, exhibiting a polydispersity index (PDI) of 0.19, whereas P2-SeNPs show a higher PDI of 0.24 (Figure S4).

#### 3.3.2. Morphological Evaluation

**Figure 5:**
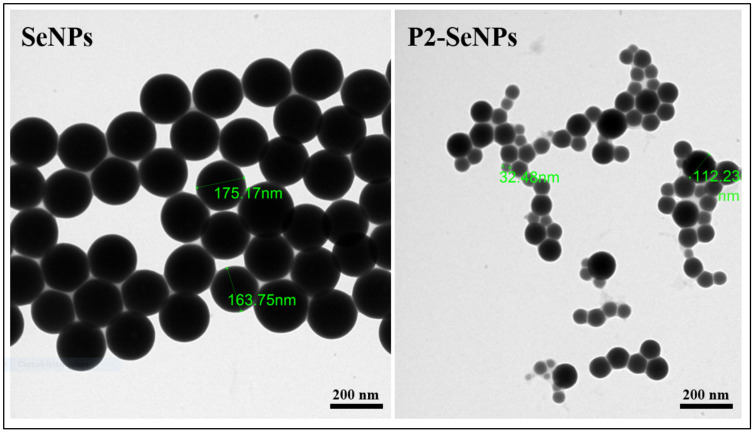
TEM images of SeNPs and P2-SeNPs.

TEM is a widely employed technique for observing the morphology of nanoparticles. As shown in Figure 5, without the addition of P2, the synthesized SeNPs exhibit a uniform distribution of particle sizes from 163 to 175 nm. In contrast, P2-SeNPs present dispersion and a smaller particle size ranging from 32 to 112 nm, compared to SeNPs. SeNPs are unstable and aggregated to a large cluster. These results highlight the essential role of P2 in stabilizing SeNPs, attributed to the abundance of terminal hydroxyl groups on polysaccharide chains, which strongly adsorb onto the SeNP surface [47]. It is important to highlight that the particle size observed by TEM is smaller than that obtained by DLS, as TEM measures the size of nanoparticles in their dry state, while DLS determines the hydrodynamic diameter, which includes both the nanoparticle core and the surrounding solvent shell. Further optimization of synthesis parameters, such as fine-tuning of P2 concentration, reaction kinetics, and sonication, may help achieve a more uniform size distribution, enhancing the suitability of P2-SeNPs for biomedical applications.

#### 3.3.3. Interactions Between P2 and SeNPs via FT-IR Spectroscopy Analysis

**Figure 6:**
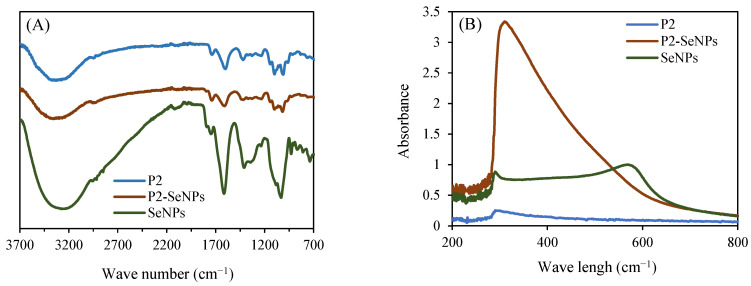
FT-IR spectra (**A**) and UV-Vis spectra (**B**) of P2-SeNPs and SeNPs.

The interaction mechanism between P2 and SeNPs was confirmed using FT-IR spectroscopy (Figure 6A). Overall, no significant changes were observed in the characteristic peaks of P2 compared to those of P2-SeNPs. However, slight variations in the stretching vibrations were noted at two specific peaks. In all spectra, the characteristic absorption peaks corresponding to hydroxyl groups (−OH stretching) were detected. Notably, the hydroxyl peak shifted from 3297 cm^−1^ in P2 to 3318 cm^−1^ in P2-SeNPs, indicating a possible hydrogen bonding interaction between the O−H groups of P2 and SeNPs [43]. The absorption peak at 1097 associated with C−O−C stretching in P2 slight shifts to 1010 cm^−1^ for P2-SeNPs, suggesting interaction of P2 with SeNPs. On the other hand, the peaks at 1409 cm^−1^ due to C−H bending and 1743 cm^−1^ characteristic of C=O stretching are observed for both SeNPs and P2-SeNPs. These results suggest that Se−O bonds could be formed between SeNPs and P2.

#### 3.3.4. UV-Vis Spectroscopy Analysis

Figure 6B presents the UV-Vis absorption spectra of P2, SeNPs, and P2-SeNPs. The spectrum of P2 alone shows very low absorption across the measured range, indicating that P2 itself does not significantly absorb in the UV-Vis region. In contrast, SeNPs exhibit a characteristic absorption peak around 300 nm, attributed to the surface plasmon resonance of colloidal SeNPs [48]. The spectrum of P2-SeNPs also shows a pronounced peak at 300 nm, but with much stronger absorbance than SeNPs alone. This increase suggests an interaction between SeNPs and P2, possibly due to enhanced dispersion or stabilization effects. Additionally, SeNPs exhibit a secondary absorption feature at around 600 nm, which is absent in P2-SeNPs, suggesting that unmodified SeNPs are more prone to aggregation and precipitation in the absence of P2.

#### 3.3.5. XRD Analysis

**Figure 7:**
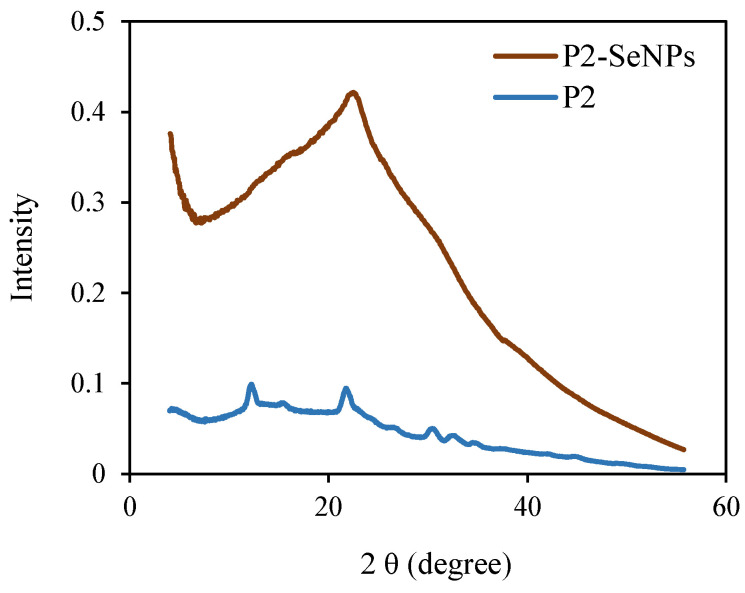
XRD patterns of P2-SeNPs and P2.

The amorphous structure and elemental state play a crucial role in the high bioactivity and low cytotoxicity of SeNPs [49]. The XRD spectrum of P2 alone exhibits two low-intensity diffraction peaks at 12.5 and 22.3° (Figure 7). This indicates that P2 is a semi-crystalline biopolymer. According to the literature, SeNPs synthesized without stabilizers exhibit a crystalline structure, with characteristic diffraction peaks at 2θ ≈ 24° and 30° [50]. However, in the XRD spectrum of P2-SeNPs, only a broad amorphous halo is observed, suggesting that the selenium nanoparticles adopt an amorphous state. This finding suggests that P2, as a surface-decorating agent, prevents the crystallization of selenium, likely by stabilizing nanoparticles in a disordered state [50]. Similar results have been reported, showing the amorphous nature of SeNPs when stabilized by polysaccharides [51].

#### 3.3.6. XPS Analysis

**Figure 8:**
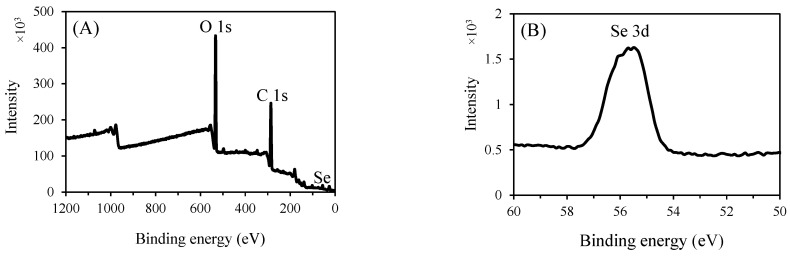
Wide-range XPS patterns and (**A**) Se 3d spectra (**B**) of P2-SeNPs.

XPS spectra were acquired to examine the valence state of selenium in P2-SeNPs. As illustrated in Figure 8A, in addition to the signals from O and C, a typical Se 3d peak is observed in the spectrum of P2-SeNPs. This Se 3d peak at 55.4 eV indicates that P2-stabilized SeNPs consist of selenium in the zero-valent state (Figure 8B). Furthermore, the spectrum of P2-SeNPs does not show a peak at 59.1 eV, which corresponds to Se IV, confirming the absence of Na₂SeO₃ in the final product [51].

Based on these observations, the formation mechanism of P2-SeNPs can be described as follows: the SeO₃^2−^ precursor is first adsorbed onto P2 chains through electrostatic interactions with the hydroxyl groups, forming a specific chain-like intermediate. Next, SeO₃^2−^ is reduced in situ to Se by ascorbic acid and then grows to yield P2-SeNPs on which P2 is adsorbed through Se–O bond interactions, preventing the coalescence and agglomeration.

### 3.4. Stability of P2-SeNPs

The stability of selenium nanoparticles in aqueous solution plays a crucial role in their biological activity and determines their applicability in various fields [52]. Since unmodified SeNPs are prone to aggregation and precipitation, P2 was used as a stabilizer. Stability was assessed by monitoring size variations under different conditions, including storage time, temperature, pH, and ionic strength.

#### 3.4.1. Storage Stability

**Figure 9:**
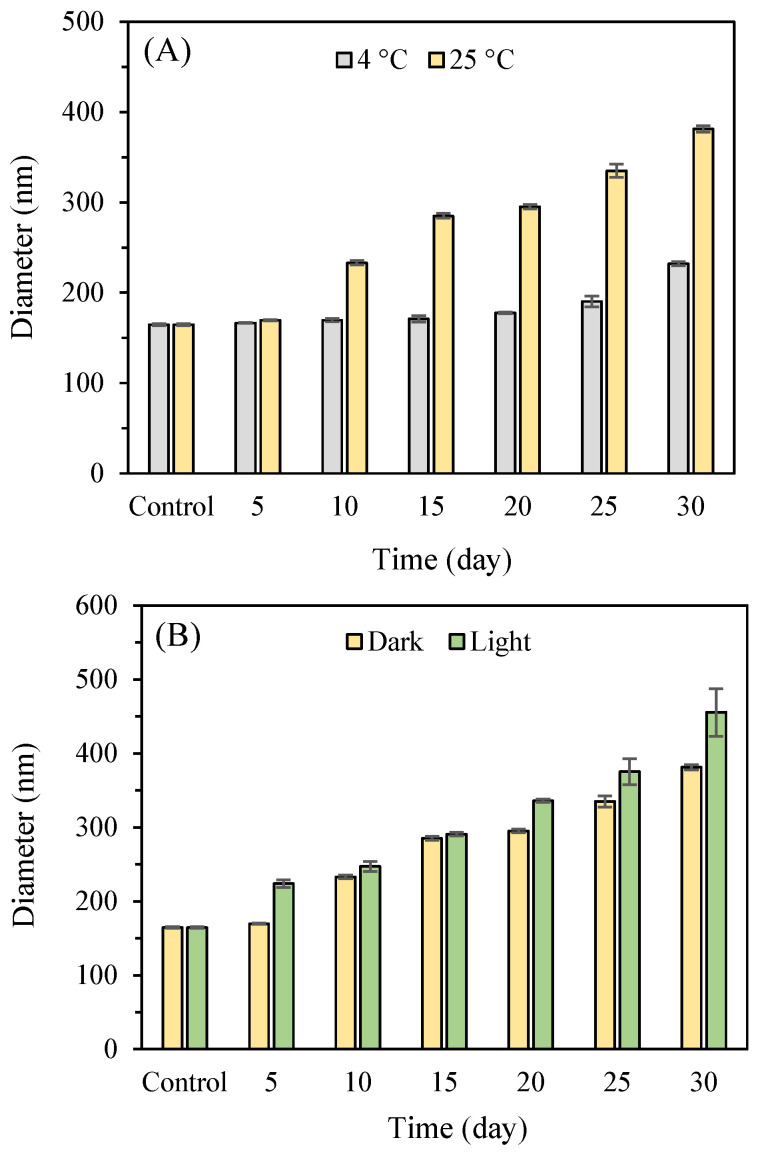
Changes in the average size of P2-SeNPs stored at 4 °C and 25 °C in darkness (**A**) and under both light and dark conditions at 25 °C (**B**) over 30 days.

To evaluate the stability of nanoparticles over an extended period, the storage time was increased to 30 days, with samples stored at both 4 °C and 25 °C, under both light and dark conditions. Figure 9A illustrates the changes in the average size of P2-SeNPs stored at these temperatures over a 30-day period in the dark. The initial average diameter of P2-SeNPs was 164.6 ± 1.1 nm. At 4 °C, the average size gradually increases over time, reaching 177.6 ± 1.1 nm et 232.2 ± 2.1 at day 20 and day 30, respectively. At 25 °C, the size increase is more pronounced, with a diameter of 295.2 ± 2.5 nm and 381.2 ± 3.4 at day 20 and day 30, respectively. The significant increase in particle size suggests that higher temperature accelerates the aggregation of nanoparticles, likely due to increased molecular motion and interaction rates. Figure 9B illustrates the variation in the size of P2-SeNPs after being stored for 30 days at 25 °C, both in light and dark conditions. The effect of light exposure was examined at 25 °C to evaluate their behavior under ambient conditions, which are more relevant for practical applications. In the presence of light, the increase in size is more significant than in the dark, reaching 455.5 ± 32.1 nm by day 30. These findings indicate that P2-SeNPs possess good stability at refrigerated temperatures and in darkness, consistent with the study by Jiang et al., which highlighted the adverse impact of higher temperatures on SeNP stability [53].

#### 3.4.2. Influence of pH and Ionic Strength

**Figure 10:**
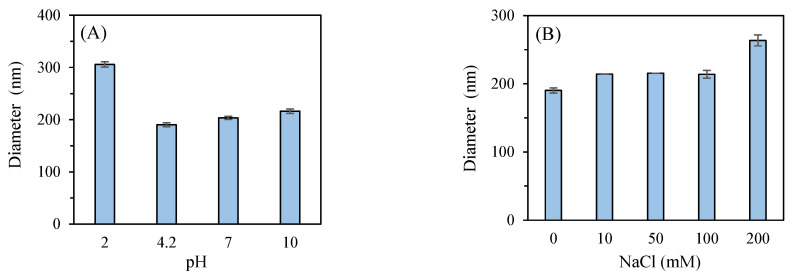
Impact of pH (**A**) and different ionic strengths (**B**) on the stability of P2-SeNPs.

The impact of pH on the stability of P2-SeNPs was studied (Figure 10A). The P2-SeNPs solution stored at 4 °C for 10 days had a pH of 4.2, a particle size of 190.3 ± 3.7 nm, and a zeta potential of −6 mV. At pH 2, the particle size increased to 305.8 ± 5.1 nm, and the zeta potential shifted to 0.9 mV, signaling a loss of electrostatic repulsion and promoting aggregation due to protonation of functional groups on the polysaccharide chains [54]. At pH 7, the size increased slightly to 203.5 ± 2.9 nm, with the zeta potential slightly decreasing to −6.4 mV, indicating moderate stability under neutral conditions. Under basic conditions (pH = 10), the particle size further increased to 216.4 ± 4.3 nm, and the zeta potential became more negative at −11.6 mV, suggesting that deprotonation of functional groups at high pH reduces electrostatic stabilization, leading to moderate aggregation. Overall, P2-SeNPs exhibit the best stability near their initial pH of 4.2, with significant destabilization occurring under strongly acidic conditions.

The effect of ionic strength was also considered (Figure 10B). At low to moderate ionic strengths (10–100 mM NaCl), the particle size slightly increases to 215.5 ± 0.1 nm, suggesting a reasonable stability of P2-SeNPs. In contrast, at high ionic strength (200 mM NaCl), the particle size significantly increases to 263.6 ± 7.9 nm, indicating substantial aggregation. A high ionic strength reduces the electrostatic repulsion between the nanoparticles, thus promoting aggregation.

### 3.5. Antioxidant Activities of Nanoparticles

The antioxidant activities of SeNPs and P2-SeNPs were assessed by measuring their ability to scavenge DPPH and ABTS radicals, as well as their metal chelation capacity. As shown in Table S1, P2-SeNPs demonstrate significantly higher DPPH radical scavenging activity (88.3%), ABTS radical scavenging activity (77.9%), and metal chelation ability (76.7%) compared to SeNPs (*p* < 0.05). The antioxidant capacity of SeNPs is known to be influenced by particle size, with smaller particles exhibiting a larger specific surface area and more reactive sites for free radicals [55]. Furthermore, the stability of SeNPs plays a crucial role in their antioxidant activities [56]. Poor stability leads to aggregation, reducing the active surface area available for interactions with free radicals. Consequently, the smaller size and improved stability of P2-SeNPs contribute to their enhanced antioxidant activities in comparison to SeNPs.

## 4. Conclusions

This study effectively established the synthesis and stabilization of selenium nanoparticles (SeNPs) using the purified polysaccharide P2, which was extracted from *O. natrix*. Characterization techniques confirmed that P2 effectively stabilized SeNPs, as indicated by smaller particle size and reduced aggregation. Evidence of the interaction between P2 and SeNPs was provided by FT-IR, XRD, and XPS analyses. Stability tests showed that P2-SeNPs retained their integrity better than unmodified SeNPs under various storage conditions, including temperature and pH variations. Additionally, P2-SeNPs exhibited enhanced antioxidant activities relative to SeNPs, with higher scavenging abilities against free radicals and improved metal chelation. These results highlight the promise of P2-SeNPs as a valuable material for antioxidant applications in biomedicine, presenting a more stable and efficient alternative to conventional SeNPs. Further studies are underway to evaluate the antioxidant activity of P2-SeNPs under various storage conditions and their cytotoxicity to assess biocompatibility and safety for biomedical applications.

## Figures and Tables

**Table 1:** Weight-average molecular weight (Mw) and dispersity (Ð) data of purified polysaccharides P1 and P2.

	Mw (kDa)	Ð
P1		
Peak 1	732.6	1.07
Peak 2	74.4	1.6
P2	30.2	1.7

## Data Availability

Data are contained within the article and Supplementary Materials.

## Supplementary Materials

The following supporting information can be downloaded at: https://www.mdpi.com/article/10.3390/nano15060435/s1, Figure S1: GPC chromatograms of purified polysaccharides P1 and P2; Figure S2: TGA thermograms of purified polysaccharides P1 and P2; Figure S3: Selenium nanoparticles SeNPs and P2-SeNPs freshly prepared (A), lyophilized (B), and stored at 4 °C for 30 days (C); Figure S4: DLS size distribution of SeNPs and P2-SeNPs; Table S1: DPPH radical scavenging activity, ABTS radical scavenging capacity, and metal chelation ability of SeNPs and P2-SeNPs.
