# Supplementary material for: Synthesis, Characterization, and Stability Study of Selenium Nanoparticles Coated with Purified Polysaccharides from Ononis natrix
Source: Nanomaterials (Basel). 2025 Mar 12;15(6):435. doi: 10.3390/nano15060435 (PMC11946226; doi:10.3390/nano15060435)
Supplement: Supplementary file 1 [file nanomaterials-15-00435-s001.zip › nanomaterials-3487913-supplementary.pdf]

nanomaterials

Supplementary Materials

Synthesis, Characterization, and Stability Study of Selenium Nanoparticles Coated with Purified Polysaccharides from *Ononis natrix*

Nour Bhiri [1,2], Nathalie Masquelez [1], Moncef Nasri [2], Rim Nasri [2], Mohamed Hajji [2,*] and Suming Li [1,*]

[1] Institut Européen des Membranes, IEM, UMR 5635, University of Montpellier, CNRS, ENSCM, 34095 Montpellier, France; nour.bhiri@enis.tn (N.B.); nathalie.masquelez@umontpellier.fr (N.M.)
[2] Laboratory of Enzyme Engineering and Microbiology, National School of Engineering of Sfax (ENIS), University of Sfax, P.O. Box 1173, Sfax 3038, Tunisia; mon_nasri@yahoo.fr (M.N.); rymnasri2@gmail.com (R.N.)
* Correspondence: hajjimed1979@yahoo.fr (M.H.); suming.li@umontpellier.fr (S.L.)

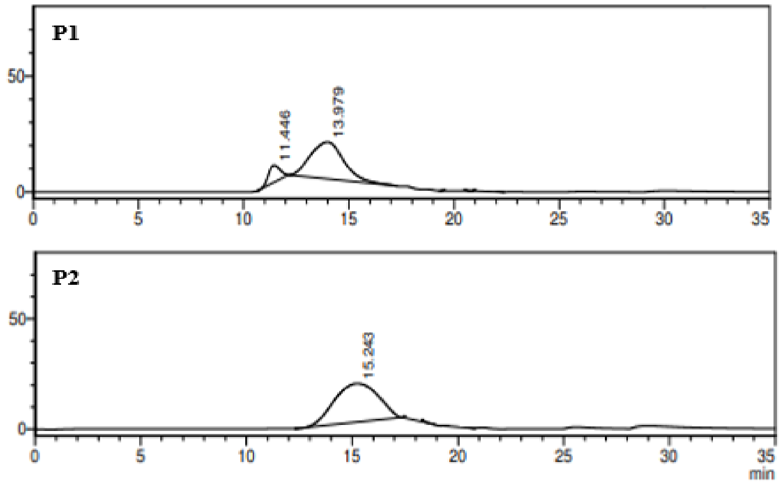

Figure S1. GPC chromatograms of purified polysaccharides P1 and P2.

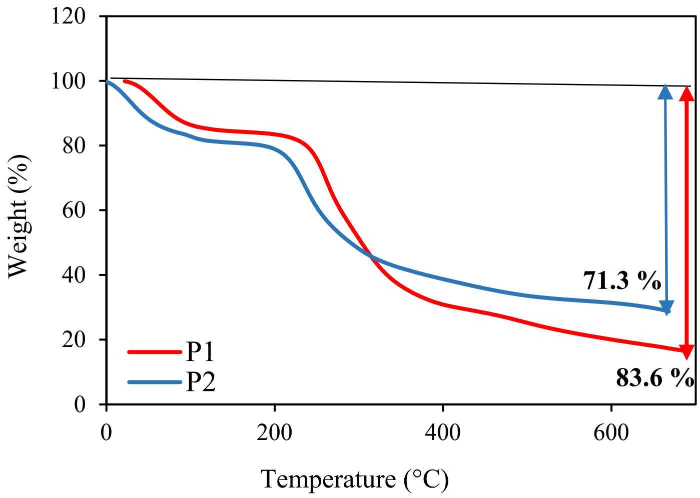

Figure S2. TGA thermograms of purified polysaccharides P1 and P2.

 https://doi.org/10.3390/nano15060435

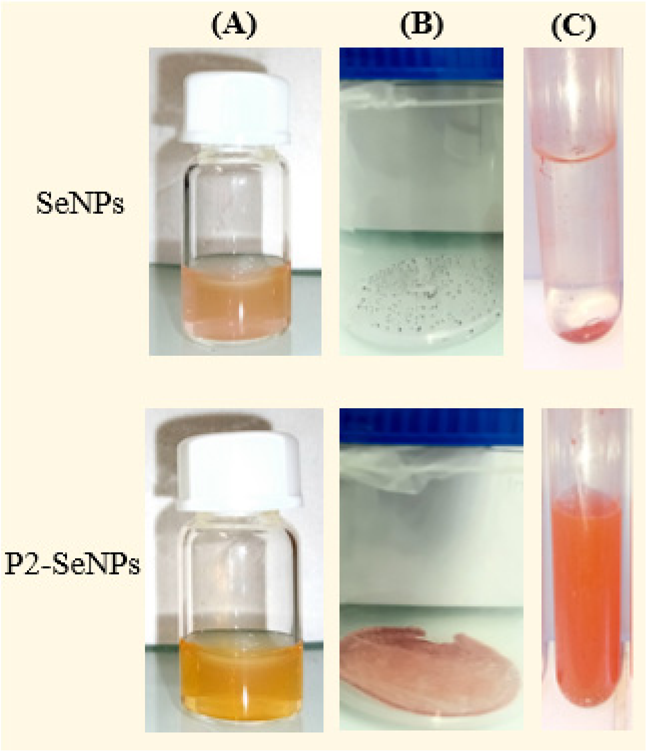

Figure S3. Selenium nanoparticles SeNPs and P2-SeNPs freshly prepared (**A**), lyophilized (**B**), and stored at 4 °C for 30 Days (**C**).

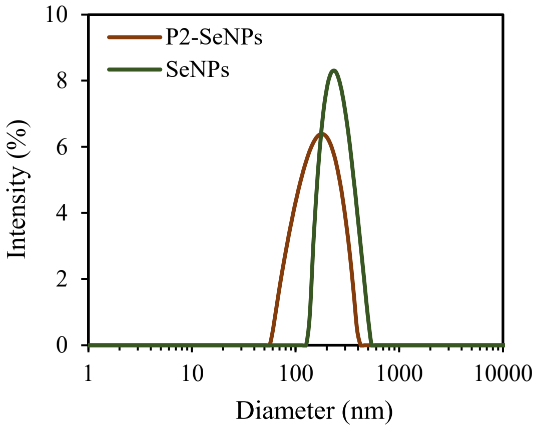

Figure S4. DLS size distribution of SeNPs and P2-SeNPs.

Table S1. DPPH radical scavenging activity, ABTS radical scavenging capacity, and metal chelation ability of SeNPs and P2-SeNPs.

	DPPH (%)	ABTS (%)	Metal chelating (%)
SeNPS	52.9±0.2	48.9±0.8	42.5±0.6
P2-SeNPs	88.3±0.5	77.9±0.4	76.7±0.3
